# Research on the Design Method of a Bionic Suspension Workpiece Based on the Wing Structure of an Albatross

**DOI:** 10.1155/2019/2539410

**Published:** 2019-02-03

**Authors:** Siyang Gao, Bangcheng Zhang, Jianwei Sun

**Affiliations:** School of Mechatronic Engineering, Changchun University of Technology, Changchun 130012, China

## Abstract

An air suspension platform uses air pressure to realize the suspension function during the suspension process, and it has the disadvantage of large air pressure and a small suspension force. In this study, an air suspension platform was built using bionic design to reduce the required air pressure and increase the suspension force. A suspension structure mapping model was established according to the physiological structure characteristics of albatross wings. A bionic model was established by using the theoretical calculation formula and structural size parameters of the structural design. A 3D printer was used to manufacture the physical prototype of the suspended workpiece. Based on this, a suspension test rig was built. Six sets of contrast experiments were designed. The experimental results of the suspension test bench were compared with the theoretical calculation results. The results show that the buoyancy of the suspended workpiece with a V-shaped surface at a 15-degree attack angle was optimal for the same air pressure as the other workpieces. The surface structure of the suspended workpiece was applied to the air static pressure guide rail. By comparing the experimental data, the air pressure of the original air suspension guide rail was reduced by 37%, and the validity of the theory and design method was verified.

## 1. Introduction

Air suspension technology is a technique that uses the buoyancy provided by flowing airflow to overcome gravity to suspend an object. This produces a gas film between two objects in contact, separating the objects and thereby reducing the friction between the two objects. Air suspension technology is widely used in bearings, guide rails, compressors, trains, blowers, glass transport equipment, metal smelting, and other aspects [1–7]. However, air suspension technology needs to provide a large airflow to complete this movement, and it is now necessary to reduce the airflow required for the work and at the same time ensure the normal operation of the air suspension workpiece. In recent years, scholars around the world have conducted a large amount of experimental research on this subject.

In the early days of research on this subject, Paivanas and Hassan developed an air output device for suspending and moving small silicon wafers (57-82 mm in diameter) based on air suspension technology, which greatly reduced the need for solid contact control with a smaller airflow device system [8]. Li et al. used gas suspension technology to design a tangential blowing suspension device, which was applied to glass transportation to improve the suspension lift of the glass while ensuring stability during the transportation of the glass [9]. Grinchuk based on the characteristics of small gas flow suspension technology to design a suspended solid particle device to save gas flow and improve the combustion efficiency of the particles [10]. Lu and Wang used air suspension technology to design an air suspension support system, which reduced the required air pressure and improved the carrying capacity of the electric platform [11]. Wei et al. proposed multi-air suspension technology, which greatly reduced the required airflow and ensured that the suspended workpiece was more stable [12].

In references [8–12], from the use of improved designs of the venting hole or control of the gas flow, under the premise of ensuring the normal operation of the equipment, the required gas pressure was reduced, and the stability of the air suspension system was guaranteed. Tian et al. designed the airfoil of small wind turbines based on the bionic swallow's extended wing, which improved the aerodynamic performance of the turbine [13]. Cheng-Yu et al. based on bionics principle to design a floating platform for the growth of the leading edge of the ear wing, changing the aerodynamic performance, improving the suspension capacity, and reducing the required air pressure [14]. Ren and Li adopted the surface structure of bionic scorpion wings, which was applied to the design of micro-aircraft to improve the upward lift during flight [15]. Ge et al. extracted biometric models from owl wings, based on bionics, and applied the models to the design of aircraft wings to improve the upward lift of the aircraft [16]. Meseguer et al. applied the frontal structure of bionic bird wings to the design of an aircraft wing, which improved the upward lift of the aircraft [17]. Widhiarini et al. applied a bionic bird wing biological model to the design of a flapping-wing micro air vehicle (FMAV) to enhance the lift of the aircraft [18]. Tian et al. used the wing structure of a bionic owl, which was applied to the design of a turbine blade, which was 12% more aerodynamic than the original turbine blade [19]. Huang based on the wing structure of a bionic eagle; a double crank rocker flapping mechanism was designed to improve the flight lift of an eagle wing [20]. In references [13–20], domestic and foreign researchers applied the principle of bionics to the design of suspension devices, effectively improving the lift of air suspension, but these designs were all used in aircraft and rotating blades, and they were not used in ordinary suspended workpieces.

To sum up, the main purpose of this paper is to design a suspended workpiece according to the flying principle and morphological structure of bionic albatross wings. The air suspension technology is used to increase the suspension force of the air suspension platform and to realize the suspension function.

Albatross wings have a special movement and flight mechanism that allows them to adapt to complex environments and fly far away. The body of the albatross is streamlined. During the flying process, the front end of the wing and the middle end of the wing are opened at a specific angle. The airflow around the wings is used to follow a boat for several hours without flapping the wings. Because albatross wings have a special surface structure, the tendons of the wings can be straightened when their wings are opened, resulting in a low probability of dynamic soaring forward and sinking when flying [21–24]. Researchers such as Richardson have introduced the unique role of the albatross in the flight mechanism and the idea that feathers are an important part of the wings of an albatross. Different feather shapes play different roles during flight. The feathers growing on the phalanx are called the primary flying feathers, which are arranged in a triangle to form the outer portion of the wing. Under the driving of the phalanx, a primary flying feather can have more free and complex movements and it is one of the main sources of the aerodynamic force [25]. The feathers growing on the forearms are called secondary feathers, and the ulnas on the inside of the wings are closely arranged into a curved surface in order to provide effective lift for flight. The feathers at the innermost layer of the root of the wing and the joint of the body are called three-stage flying feathers. These feathers form a smooth transitional aerodynamic surface, allowing the air to flow smoothly and effectively reducing the air resistance. The cover feathers on the outside of the body and on the upper surface of the wings are divided into the main cover feathers, the mid-covered feathers, and the small cover feathers, which can make the birds streamlined and increase the upward force. Each flying feather consists of a tough and elastic vane from the feather shaft and the feather piece. The upper surface of the entire wing is a streamlined curved surface. When the air flows through the upper and lower surfaces, it can produce a pressure difference between the vertical wing and the upper surface, which provides lift for the flight of the albatross. The wings of the albatross are mainly tightly buckled and arranged in an orderly manner with grooves on the surface. An albatross can lift upwards with its wings using the air to achieve dynamic soaring in the sky [26, 27]. Its excellent aerodynamic performance is related with the special structure of its wing surface. Many internal and external scholars had carried out a large number of experiments and research on the flight principle of the albatross, which solved many engineering problems. Gottfried Sachs based on the albatross flight principle to propose a new mathematical approach, which deals with the complex flight manoeuvre of dynamic soaring [28]. Richardson designed the dynamic soaring drone based on the principle of the albatross dynamic soaring, which increases the dynamic soaring distance of the dynamic soaring drone [29]. Based on the albatross flight principle, Renaud Barate designed a controller to improve the robustness of the controller [30]. Vincent Bonnin based on the flight technology principle of albatrosses to solve the problem that small UAVs face serious limitations in energy storage choice [31].

The first section of this paper introduces the current research status. The second section introduces the bionic model of the albatross wing structure. The third section mainly introduces the structure bionic model of the albatross wing angle of attack. In the fourth section, the surface structure of the bionic albatross wings was experimentally compared by setting up a suspension test rig, and the bionic wing surface structure was applied to the air static pressure guide to prove the feasibility of the theory. In the fifth section, the design theory and the experimental results of the bionic design suspended workpiece are summarized.

## 2. Albatross Wing Structure Bionic Model

### 2.1. Wing Surface Biometric Extraction and Mapping

**Figure 1:**
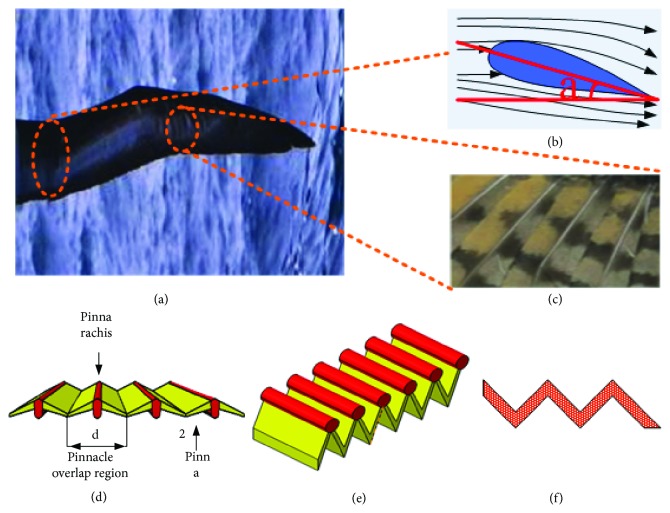
Wing surface physiological structure schematic.

The albatross wings are shown in Figure 1(a) [29]. During the flight of an albatross, there is a specific angle between the wings and the wind. This angle is called the angle of attack. The angle of attack can provide lift for the albatross during flight. A schematic diagram of the wing section is shown in Figure 1(b). The surface structure of the albatross wings is shown in Figure 1(c) [32]. The wing surface structure is a very complex structure that provides lift and thrust to flying creatures. The sliding of the bionic wings requires a detailed study of their surface structure and their motion characteristics. The wing surface structure includes the feather shaft and the feather piece. The surface structure of a wing has an important role during the flight of an albatross. Its wing surface structure is a special physiological structure, and the surface feather area presents a radial nonsmooth shape, which is mainly formed by the mutual flying feathers interlocking with each other and showing a concave-convex groove. The physiological structure diagram is shown in Figure 1(d). The symbol 1 indicates the feather shaft, the symbol 2 indicates the feather piece, and the symbol d indicates the overlapping area of the feather piece. Based on the surface characteristics of the wing structure, this study simplifies its structure and extracts features. As shown in Figure 1(e), the structure diagram is shown in Figure 1(f).

**Figure 2:**
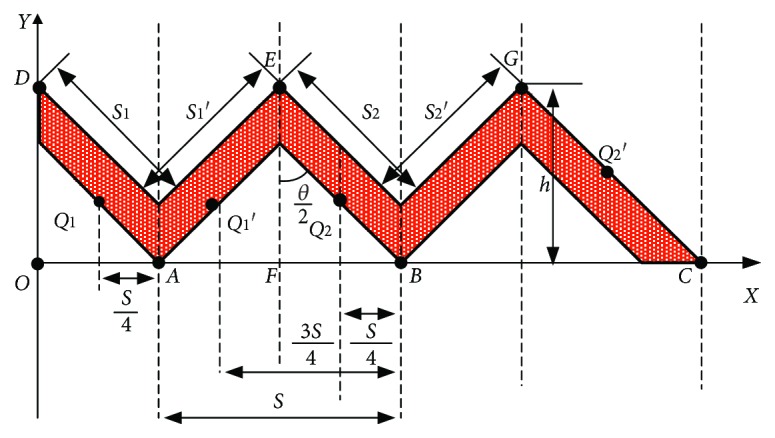
Suspended workpiece schematic.

As shown in Figure 1(f), the surface structure of the albatross wings is a concave-convex groove structure. According to the unique physiological structure of the wing shaft and the feather piece of the albatross wings, a design method was provided for the theoretical study of the bionic suspension workpiece. A schematic diagram of the surface structure of the suspended workpiece was then designed, as shown in Figure 2.

As shown in Figure 2, the fixed coordinate system is *O*_*xy*. The intersection of the surface structure of the suspended workpiece and the *X* axis is represented by *A*, *B*, and *C*, and the intersection with the *Z* axis is represented by *D*. Taking points *E*, *F*, and *G*, where EF is the midline of the triangle *AEB*, and taking *Q*_1_, *Q*_1_′, *Q*_2_, and *Q*_2_′ as the midpoints of *AD*, *AE*, *BE*, and *GC*, respectively, the distance from the projection of *Q*_1_ on the *X* axis to point *A* is 1/4*s*, the distance from the projection of *Q*_1_′ on the *X* axis to point *A* is 3/4*s*, the distance from the projection of *Q*_2_ on the *X* axis to point *B* is 1/4*s*, and the distance from the projection of *Q*_2_′ on the *X* axis to point *C* is 1/4*s*, |*AB*| = *s*, |*BE*| = *l*. The distance from point *E* to the *X* axis is *h*. *S*_1_ represents the area on the right side of the first lap, *S*_1_′ represents the area on the left side of the first lap, *S*_2_ represents the area on the right side of the second lap, *S*_2_′ represents the area on the left side of the second lap, and *n* is the number of triangular threads.

According to the geometric relationship of Figure 2,

(1) $$\begin{array}{c} l=\frac{h}{\cos\theta /2}, \end{array}$$

(2) $$\begin{array}{c} S_{1}=2\pi r_{1}l=2\pi \frac{s}{4}l, \end{array}$$

(3) $$\begin{array}{c} S_{2}=2\pi r_{2}l=2\pi \left(\frac{s}{4}+s\right)l, \end{array}$$

(4) $$\begin{array}{c} S_{n}=2\pi r_{n}l=2\pi \left(\frac{s}{4}+\left(n+1\right)s\right)l=\frac{\pi sl}{2}+2\pi sl\left(n-1\right), \end{array}$$

(5) $$\begin{array}{c} S_{k1}=s_{1}+s_{2}+\cdot \cdots \cdot +s_{n}=\pi sl\left(n^{2}-\frac{1}{2}n\right). \end{array}$$

From Equations (1) and (5),

(6) $$\begin{array}{c} S_{k1}=\frac{\pi sh}{\cos\theta /2}\left(n^{2}-\frac{1}{2}n\right). \end{array}$$

Using the same principle,

(7) $$\begin{array}{c} S_{1}^{\prime }=2\pi r_{1}^{\prime }l=2\pi \frac{3s}{4}l, \end{array}$$

(8) $$\begin{array}{c} S_{2}^{\prime }=2\pi r_{2}^{\prime }l=2\pi \left(\frac{3s}{4}+s\right)l, \end{array}$$

(9) $$\begin{array}{c} S_{n}^{\prime }=2\pi r_{n}^{\prime }l=2\pi \left[\frac{3s}{4}+\left(n-1\right)s\right]l=\frac{3\pi sl}{2}+2\pi sl\left(n-1\right), \end{array}$$

(10) $$\begin{array}{c} S_{k2}=s_{1}^{\prime }+s_{2}^{\prime }+\cdot \cdots \cdot +s_{n}^{\prime }=\pi sl\left(n^{2}-\frac{1}{2}n\right). \end{array}$$

From Equations (1) and (10),

(11) $$\begin{array}{c} S_{k2}=\frac{\pi sh}{\cos\theta /2}\left(n^{2}-\frac{1}{2}n\right). \end{array}$$

From Equations (6) and (11),

(12) $$\begin{array}{c} S=s_{k1}+s_{k2}=\frac{2\pi sl}{\cos\theta /2}n^{2}. \end{array}$$

For the condition when the upward air pressure is *P*, introducing the buoyancy coefficient *k* = 1.5, the receiving upward buoyancy *F*_1_ is obtained.

(13) $$\begin{array}{c} F_{1}=PS. \end{array}$$

From Equations (12) and (13),

(14) $$\begin{array}{c} F_{1}=k\frac{2P\pi sh}{\cos\theta /2}n^{2}. \end{array}$$

**Figure 3:**
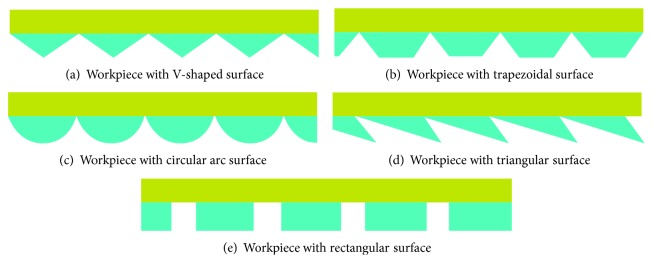
Bionic optimization structure diagram schematics.

According to the surface structure of the albatross wings, five kinds of bionic structures were designed. The structure diagrams are shown in Figure 3.

For the workpieces with V-shaped, trapezoidal, circular, triangular, and rectangular surfaces, the depth (h), width (s), and aspect ratio (h/s) are 0.25 ± 0.02 mm, 2 ± 0.02 mm, and 0.25, respectively.

**Figure 4:**
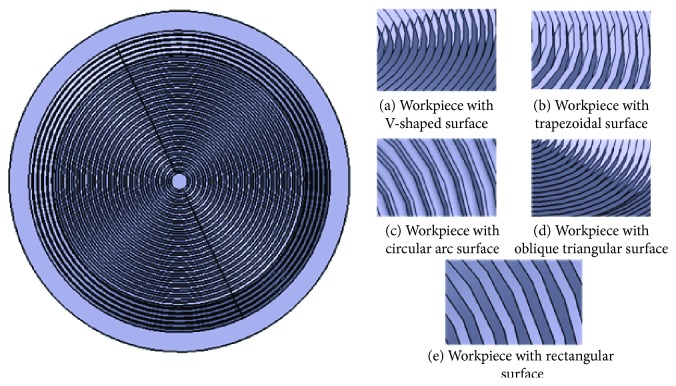
Bionic model.

The surface structures of the five bionic wings were designed to be circular and modeled by three-dimensional software, as shown in Figure 4.

### 2.2. Experimental Test

A filter regulator is a device that adjusts the air pressure value. An air suspension test stand is a device used for experimental testing. A multimeter is a device that detects the current. A balancing weight is a device for testing the mass of suspended workpieces. The trachea is used to transport gases. The battery provides electrical energy to the experimental test set.

**Figure 5:**
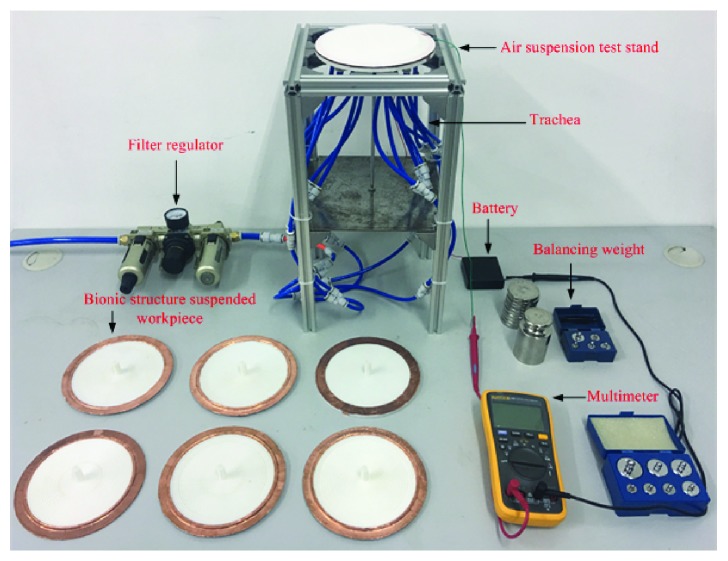
Suspended workpiece.

A 3D printer was used to print six kinds of suspended workpieces with V-shaped, trapezoidal, circular arc, rectangular, smooth plane, and oblique triangle surfaces. When doing the suspension test, the trachea is connected at the left end of the filter regulator to the air pump and the multimeter switch is turned on. The suspended workpiece is placed on the suspension test bench, the pressure value of the filter regulator is adjusted, and weights of different masses to the suspended workpiece are continuously added until the current reading on the multimeter proves that the suspended workpiece stops floating and records the mass of the weight. Six kinds of suspended workpieces were subjected to comparative experiments according to the above experimental steps, and experimental data were recorded. The experimental bench and the suspended workpieces are shown in Figure 5.

**Figure 6:**
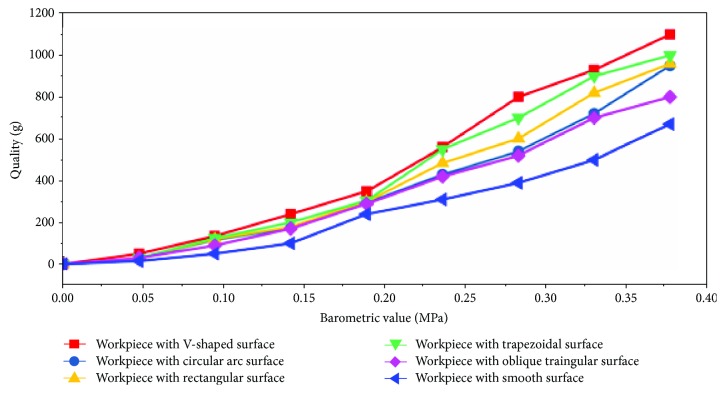
Floating workpiece data comparison chart.

The comparison experiments were carried out on the built suspension test device using the above six kinds of suspended workpieces. The experimental results are shown in Figure 6.

For the above comparative experiments, eight sets had the same air pressure. The suspended workpiece with a V-shaped surface had a larger suspension force than a circular-shaped surface suspended workpiece, a trapezoidal surface suspended workpiece, a rectangular surface suspended workpiece, an inclined triangular surface suspended workpiece, or a smooth surface suspended workpiece.

## 3. Structural Bionic Model of the Albatross Wing Angle of Attack

### 3.1. Wing Angle Feature Extraction and Mapping

**Figure 7:**
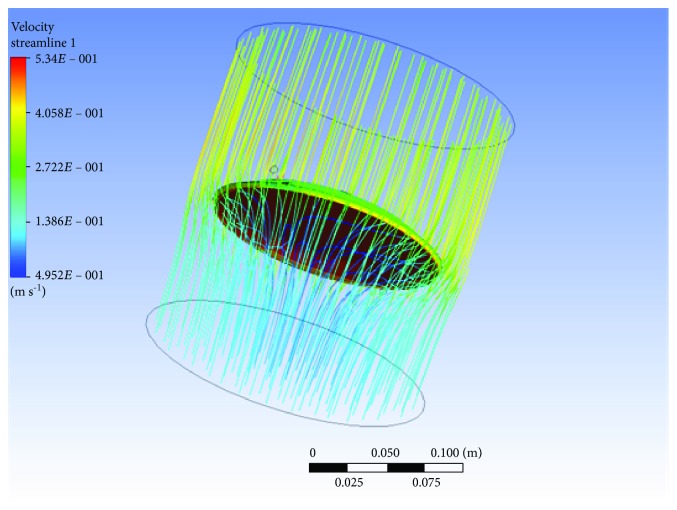
Suspended workpiece flow diagram with an angle of attack.

The airflow blows on a circular workpiece from the bottom to the top. When the airflow is blown on the lower surface of the suspended workpiece, the airflow overflows to both sides. The structure of the suspended workpiece is designed with an angle of attack to improve the upward buoyancy of the suspended workpiece. The FLUENT simulation software program is used to simulate the effect of the airflow on the suspended workpieces, as shown in Figure 7.

**Figure 8:**
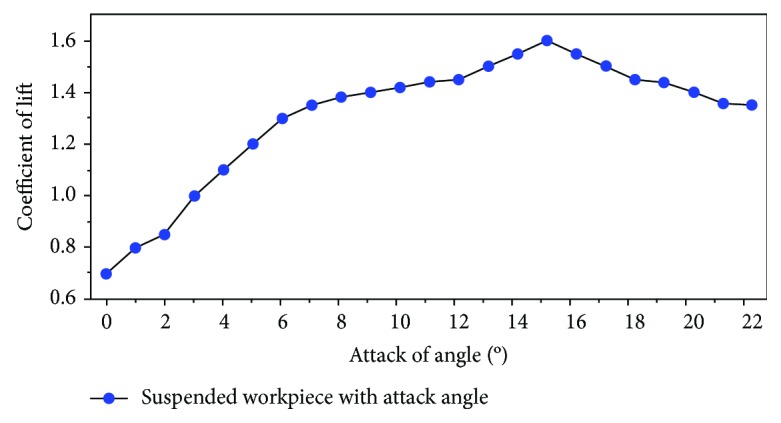
Relationship between angle of attack and lift coefficient.

According to the characteristic of the angle of attack with lift coefficient, the (CFD) method of computational fluid dynamics is used to simulate the suspension workpiece with the angle of attack. The relation between the angle of attack and the lift coefficient is obtained, as shown in Figure 8.

It can be concluded from Figure 8 that the lift coefficient does not increase with the increase of the angle of attack, but rather decreases with the increase of the angle of attack after reaching a maximum value. The calculation equation of the lift is shown below:

(15) $$\begin{array}{c} F_{2}=\frac{1}{2}{\rho \nu }^{2}{\text{s}}^{\prime }{\text{c}}_{\text{L}}, \end{array}$$

(16) $$\begin{array}{c} S^{\prime }=2\pi rn, \end{array}$$

(17) $$\begin{array}{c} F_{2}=n\frac{1}{2}{\rho \nu }^{2}2\pi r{\text{c}}_{\text{L}}. \end{array}$$

From Equations (14) and (17), the buoyancy formula of a circular workpiece with a V-shaped surface with an angle of attack is

(18) $$\begin{array}{c} F^{\prime }=k\frac{2\pi {shpn}^{2}}{\cos\theta /2}+\frac{1}{2}n{\rho \nu }^{2}{\text{s}}^{\prime }{\text{c}}_{\text{L}}. \end{array}$$

*F*′ is the buoyancy of a circular workpiece with a V-shaped surface with an angle of attack, *F*_2_ is the lift of the edge of a circular workpiece with an angle of attack, *ρ* is the air density, *ν* is the dynamic pressure, *s*′ is the reference area, and *c*_L_ is the lift coefficient.

### 3.2. Experiment Analysis

**Figure 9:**
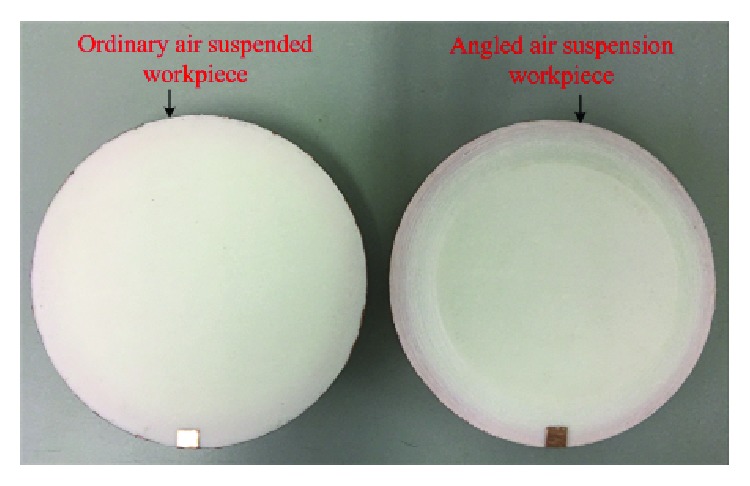
Ordinary suspension workpiece and suspended workpiece with angles of attack.

A 3D printer was used to print a suspended workpiece with no angle of attack and a suspended workpiece with an angle of attack in the built test bench (Figure 5) for experimental comparison. The suspended workpiece is shown in Figure 9.

**Figure 10:**
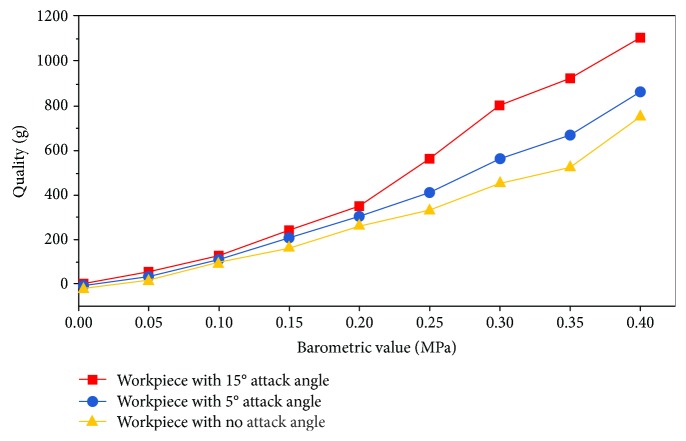
Suspended workpiece data comparison chart.

The suspended workpiece with no angle of attack and the suspended workpiece with a 15-degree angle of attack were compared on the built suspension experimental device (Figure 5). The experimental results are shown in Figure 10.

**Figure 11:**
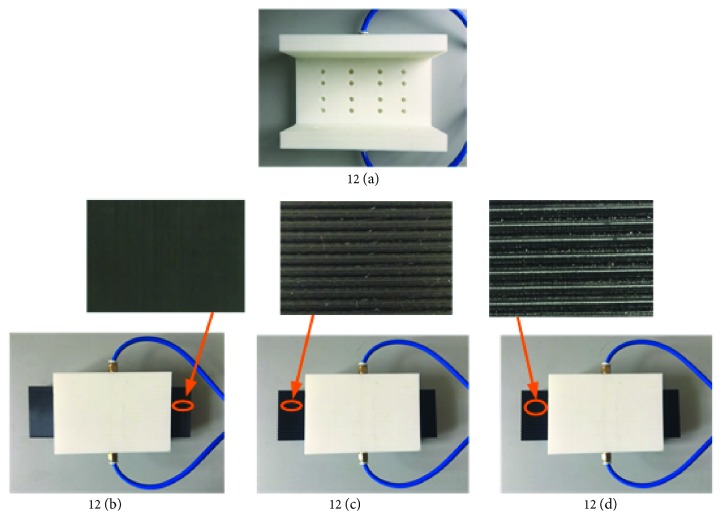
Air static pressure rail.

As shown in Figure 11, for eight sets with the same air pressure, the suspended workpiece with a 15° angle of attack has a larger suspension lift than a suspended workpiece with a 5° angle of attack and a suspended workpiece with no angle of attack.

## 4. Air Suspension Guide Bionic Manufacturing

The air suspension guide rail is mainly composed of a guide rail and a slider matched with the structure. The working principle is that the airflow is sprayed through the air outlet hole on the guide rail or the slider, and a stable air film is formed between the guide rail and the surface of the slider to suspend the slider on the guide rail, thereby reducing or eliminating contact friction between the slider and the guide rail. The motion of the slider is close to an ideal frictionless motion, a smooth movement without friction and vibration is achieved, and the advantages are high motion precision and clean and pollution-free action.

Based on the surface structure of the bionic albatross wings, the surface of the guide rail was designed into different structures for experimental testing. The slider structure is shown in Figure 11(a), and the ordinary air static pressure guide rail is shown in Figure 11(b). The guide rail surface designed as the arc-shaped structure of the 15° angle of attack is shown in Figure 11(c), and the guide rail surface designed as the V-shaped structure of 15° angle of attack is shown in Figure 11(d).

**Figure 12:**
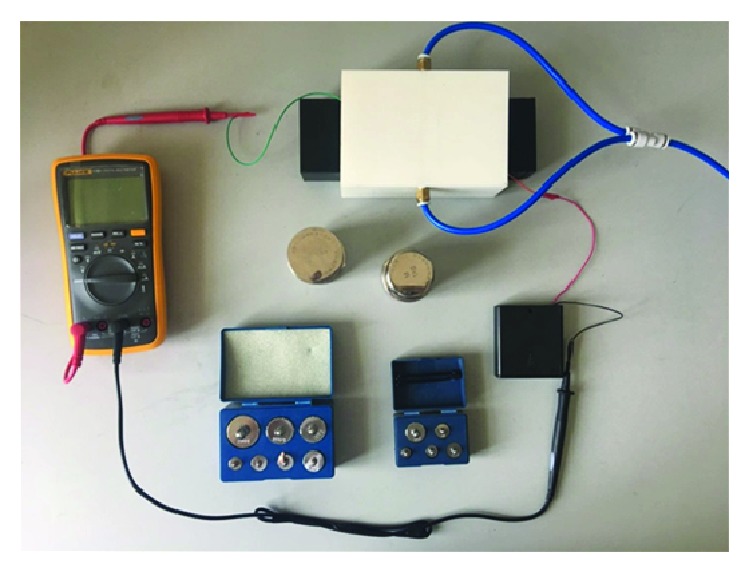
Air static pressure rail suspension device.

**Figure 13:**
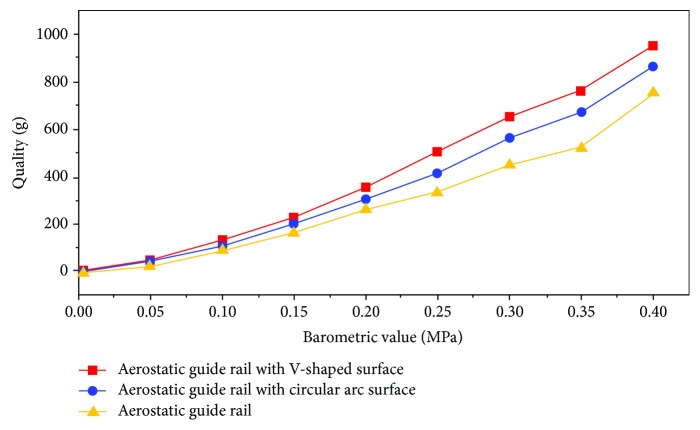
Air static pressure rail data comparison chart.

A comparison of the suspension experiments of two kinds of aerostatic guide rails on the built air static pressure rail suspension device was completed (Figure 12). A data analysis was performed on the V-shaped surface guide rail and the ordinary surface guide rail, and the experimental data comparison effect diagram was obtained, as shown in Figure 13.

As shown in Figure 13, under the same pressure for eight groups, the surface of the guide rail for a 15° angle of attack V-shaped air static pressure guide had a larger suspension force than the surface of the guide rail for a 15° angle of attack arc-shaped air static pressure guide and for an ordinary air static pressure guide rail. By designing the bionic structure of the rail surface under the same pressure, the levitation force of the aerostatic rail increased by 37%.

**Figure 14:**
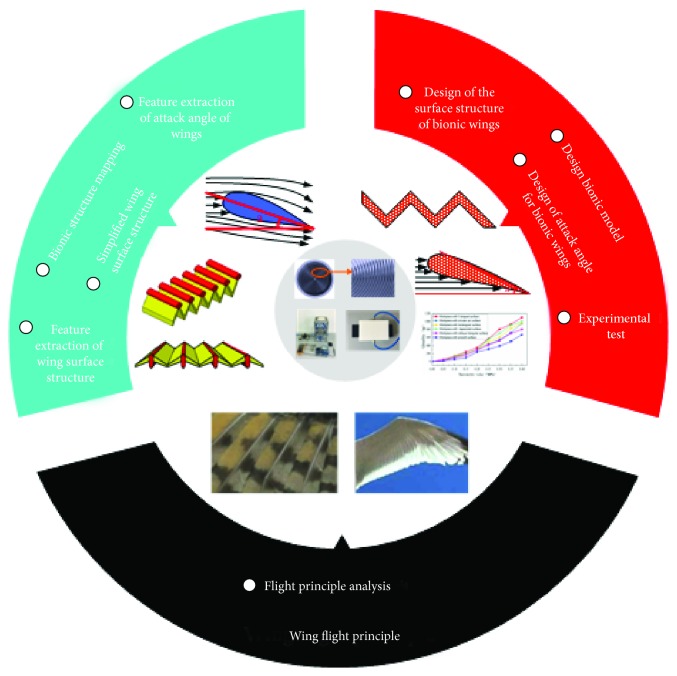
Technical route.

Based on the above research contents and methods, the technical route of the air static pressure rail is as shown in Figure 14.

## 5. Conclusion

This study built a biomapping model based on the physiological structure of albatross wings. The surface structure of the suspended workpiece, the determined structural size parameters, and the suspension coefficient were designed based on the structural characteristics of albatross wings.

This study designed six kinds of bionic suspension workpieces. Suspension comparison experiments were carried out, and experimental data was gathered using the designed test rig for the same pressure conditions for eight groups. The suspended workpiece with a V-shaped surface had a larger suspension force than the circular-shaped surface suspended workpiece, the trapezoidal surface suspended workpiece, the rectangular surface suspended workpiece, the inclined triangular surface suspended workpiece, and the smooth surface suspended workpiece.

In this study, based on the bionic mapping design, the surface structure of the suspended workpiece is applied to the air static pressure rail. The result shows that the air pressure required for the normal operation of the air static rail can be reduced by 37%.

## Data Availability

The data used to support the findings of this study are available from the corresponding author upon request.
